# Per- and Polyfluoroalkyl Substances and Breastfeeding as a Vulnerable Function: A Systematic Review of Epidemiological Studies

**DOI:** 10.3390/toxics11040325

**Published:** 2023-03-29

**Authors:** Amalie Timmermann, Oyemwenosa N. Avenbuan, Megan E. Romano, Joseph M. Braun, Janne S. Tolstrup, Laura N. Vandenberg, Suzanne E. Fenton

**Affiliations:** 1 National Institute of Public Health, University of Southern Denmark, 1455 Copenhagen, Denmark; 2 Curriculum in Toxicology and Environmental Medicine, University of North Carolina, Chapel Hill, NC 27 599-7325, USA; 3 Department of Epidemiology, Dartmouth Geisel School of Medicine, Hanover, NH 03 755, USA; 4 Department of Epidemiology, Brown University, Providence, RI 02 903, USA; 5 Department of Environmental Health Sciences, School of Public Health and Health Sciences, University of Massachusetts Amherst, Amherst, MA 01 003, USA; 6 Mechanistic Toxicology Branch, Division of the National Toxicology Program, National Institute of Environmental Health Sciences, Durham, NC 27 709, USA

**Keywords:** endocrine disrupting chemical, lactation, nursing, perfluoroalkyl substances, PFAS, primiparous

## Abstract

Milk formation in the breast during breastfeeding is a complex hormonally regulated process, potentially sensitive to the effects of endocrine-disrupting chemical exposures. The environmental chemicals, per- and polyfluoroalkyl substances (PFAS) are known endocrine disruptors. PFAS exposure have been associated with insufficient mammary gland development in mice and reduced breastfeeding duration in humans. The aim of this review was to gather the epidemiological evidence on the association between PFAS exposure and breastfeeding duration. Using PubMed and Embase, we performed a systematic literature search (on 23 January 2023) to identify epidemiological studies examining the association between maternal PFAS exposure and breastfeeding duration. Animal studies, reviews, and non-English studies were excluded. The risk of bias was assessed using the risk of bias in non-randomized studies of exposures tool. Estimates describing the association between PFAS exposure and the duration of breastfeeding were identified, and the data were synthesized separately for each type of PFAS and for the duration of exclusive and total breastfeeding. Six studies with between 336 and 2374 participants each were identified. PFAS exposure was assessed in serum samples (five studies) or based on residential address (one study). Five out of six studies found shorter total duration of breastfeeding with higher PFAS exposure. The most consistent associations were seen for perfluorooctane sulfonate (PFOS), perfluorooctanoic acid (PFOA), and perfluorononanoic acid (PFNA). The finding of a potential causal association between PFAS exposure and breastfeeding duration is in agreement with findings from experimental studies.

## 1. Introduction

According to the World Health Organization, “breastfeeding is one of the most effective ways to ensure child health and survival” [1]. Breastmilk components include micronutrients and immunoglobulins, which shape the infant’s immune development [2,3], and breastfeeding has been associated with a reduced risk of respiratory infections [4], infection-related and all-cause mortality [5], attention deficit/hyperactivity disorder [6], and acute lymphoblastic leukemia [7], as well as higher intelligence [8] in the child. Maternal benefits associated with breastfeeding include reduced risk of breast cancer, ovarian cancer, and type 2 diabetes [9,10]. The World Health Organization and the American Academy of Pediatrics recommend exclusive breastfeeding for the first six months of infancy and continued breastfeeding for a year or more [11,12]. Yet, in low- and middle-income countries, less than 40% of infants younger than 6 months were breastfed [9]. In high-income countries breastfeeding duration is generally even shorter [9], and women often report experiences of undesired early weaning due to inadequate milk supply [13,14,15,16,17,18]. Sufficient milk formation depends on the development of the breast during early life, puberty, and pregnancy, a process which is hormonally regulated [19] and, subsequently, sensitive to effects of endocrine disrupting chemical exposures.

Per- and polyfluoroalkyl substances (PFAS) are a group of environmentally persistent oil and water-repellent chemicals widely used in consumer products such as food packaging, cosmetics, clothing, and furniture upholstery [20,21]. Human exposure to PFAS occurs through contaminated food and water, as well as inhaled exposures from treated materials or point sources [22]. For many years, the most widely produced and used types of PFAS were perfluorooctane sulfonate (PFOS) and perfluorooctanoic acid (PFOA), but after they were listed under the Stockholm Convention on persistent organic pollutants in 2009 and 2019, respectively [23], they have been gradually replaced in global industrial products by other types of PFAS, including, but not limited to, perfluorohexane sulfonate (PFHxS), which was listed under the Stockholm Convention in 2022 [23], perfluorononanoic acid (PFNA), perfluorodecanoic acid (PFDA), perfluoroundecanoic acid (PFUnDA), perfluorododecanoic acid (PFDoDA), perfluoroheptane sulfonate (PFHpS), and perfluorotridecanoic acid (PFTrDA), and others.

PFAS are known endocrine disruptors, with reported effects on female reproductive outcomes [24], and they have multiple other adverse health effects, including effects on the immune system, thyroid function, liver disease, kidney disease, cancer, lipid and insulin regulation, and developmental outcomes [25]. PFAS are transferred with breastmilk [26,27,28,29,30] and have been shown to have immunotoxic effects in children [31,32], especially during early infancy [33].

Early studies regarding the health effects of PFOA demonstrated a dose-dependent morbidity and mortality in mouse pups the first few days after birth [34]. A year later, those prenatal PFOA-induced effects were shown to be due to insufficient development of the dam mammary gland during pregnancy and deficient milk production during lactation [35]. In the last decade, several well-powered and well-designed cohort studies examining the association between PFAS and breastfeeding duration in women have been published. The aim of this systematic literature review was to summarize the effect measures describing the associations between maternal PFAS exposure and duration of breastfeeding across epidemiological studies, while taking differences in methodology and potential study-related bias into account.

## 2. Materials and Methods

On 19 January 2022, we performed a systematic literature search using PubMed and Embase (Ovid) to identify epidemiological studies examining the association between PFAS exposure in pregnant or breastfeeding women and the, subsequent, duration of breastfeeding. The search was repeated on 19 August 2022 and 23 January 2023. The search consisted of two facets, and the search terms within each facet were separated by an ‘OR’ operator, while the two facets were combined using an ‘AND’ operator (Table 1). Facet 1 consisted of different terms relevant to PFAS, including abbreviations of two of the most often detected/measured PFAS, namely PFOA and PFOS. Most studies on PFAS will include at least one of the most commonly reported PFAS, and by including PFOA and PFOS in the search we aimed to find studies that might not have used the collective term PFAS. Older studies often referred to PFAS as perfluorinated chemicals (PFC), but PFC is a more restrictive term and may not encompass all the PFAS reported. Facet 2 covered breastfeeding either as part of the title or as a key term. The search was restricted to records in English, and reviews were excluded (Table 1).

The obtained records were imported into EndNote X9 (Clarivate, Chandler, AZ, USA), and duplicates were removed. One author (AT) performed the title and abstract screening and recorded the reasons for exclusion (biomonitoring reports, studies of PFAS transfer, studies of other outcomes than breastfeeding, reviews, and animal studies). All the records that were not excluded were read in full and assessed for eligibility, and those deemed eligible (epidemiological studies examining associations between maternal PFAS exposure and the duration of breastfeeding) were included in the review, in accordance with the PECO statement (Table 2), which describes the population, exposure, comparator, and outcomes [36].

Key information from the selected studies were gathered using Microsoft Excel version 16 (Microsoft Corporation, Redmond, WA, USA). The following key data were collected: first author, year of publication, study country, study design, number of participants (based on the largest number of participants in the main analyses), methods for assessing PFAS exposure and breastfeeding duration, median PFAS concentrations and duration of breastfeeding, statistical methods, adjustment set, approach to confounding from previous breastfeeding, effect estimates, and author conclusion.

The quality of the studies was assessed by two authors in collaboration (OA and AT) using the risk of bias in non-randomized studies of exposures (ROBINS-E) tool [37]. ROBINS-E was designed to assess the potential impact of an exposure on an outcome in non-randomized studies, according to seven domains: (1) risk of bias due to confounding, (2) risk of bias arising from measurement of the exposure, (3) risk of bias in the selection of participants in the study (or in the analysis), (4) risk of bias due to post-exposure interventions, (5) risk of bias due to missing data, (6) risk of bias arising from measurement of the outcome, and (7) risk of bias in the selection of the reported result. For each domain, the risk of bias was assessed as low risk, some concerns, high risk, or very high risk of bias [37]. In observational studies, the risk of residual confounding can seldom be excluded, and when assessing risk of bias due to confounding using ROBINS-E, low risk of bias is thus interpreted as a low risk of bias, except for concerns about residual confounding [37].

We assessed whether each study used appropriate methods to control for confounding, such as (but not exclusive to) adjustment, stratification, and restriction. Potential confounders relevant to most or all of the studies were reviewed. These factors included: prior history of breastfeeding, parity, maternal age, body mass index (BMI), gestational age, and education, but potential confounding factors will differ across studies, based on the study design, the PFAS exposure routes, and the reasons for breastfeeding termination. We considered potential confounding from previous breastfeeding particularly important, since PFAS are transferred to the infant in breastmilk [26,27,28,29,30], and thus, breastfeeding decreases maternal serum PFAS concentrations [38]. Consequently, women who have previously breastfed for a prolonged period will have lowered their circulating PFAS concentrations, and they may also breastfeed their next child and for a longer period [39]. Mothers that have not breastfed their previous children or only breastfed for a short period of time, will have relatively higher blood PFAS concentrations, and may be more likely to terminate breastfeeding of their next child early, resulting in a non-causal association between PFAS concentrations and the duration of breastfeeding.

When assessing bias due to exposure or outcome measurement errors we focused on the risk of differential misclassification. PFAS exposure can be measured using biological material or by using proxy measures. The precision of each method depends on the circumstances in the specific study, but some imprecision is inevitable. However, if the misclassification is non-differential, bias will most likely be towards null.

Bias due to missing data was assessed by identifying the extent of missing information about the exposure, outcomes, and potential confounders. If missingness was likely to affect the results, we further assessed the measures taken to address the bias.

The overall risk of bias was created using an algorithm based on all the domains of ROBINS-E. For a study to receive an overall low risk of bias assessment, the study would have to rank as low risk of bias in each query domain. This means that aside from the possibility of uncontrolled confounding, inherent to the study’s observational nature, there is little or no concern of bias in the study results.

The effect measures of interest were those describing the association between estimates of PFAS exposure and reported duration of breastfeeding, or the risk of breastfeeding termination at a given timepoint. The results were synthesized separately for each type of PFAS and for the duration of exclusive and total breastfeeding, with the duration of exclusive breastfeeding being the period from birth until introduction of formula or solid foods, and total duration of breastfeeding being the period from birth until breastfeeding is terminated completely. Two of the included studies reported results on breastfeeding initiation, and these endpoints were added to Section 3 after the initiation of the review. Due to the diversity of the included studies, it was not possible to identify a standardized metric to use across studies, and we, thus, reported the results based on the metrics used in each study.

## 3. Results

**Figure 1:**
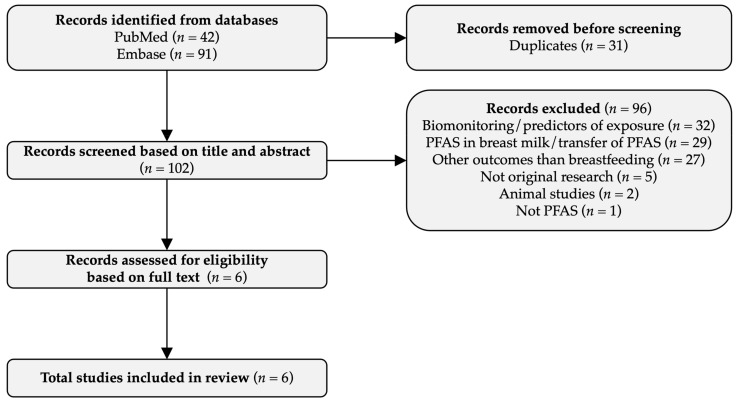
Flow diagram.

In the initial search in January 2022, 108 records were identified, 17 additional records were identified in August 2022, and 8 additional records were identified in January 2023. Among the 133 records identified in the most recent search, 31 were duplicates (Figure 1), and the majority of the remaining records did not examine the effects of PFAS on breastfeeding, but rather the PFAS concentrations in breastmilk, the transplacental transfer of PFAS from mother to child, the predictors of PFAS concentrations, or the PFAS associations with other outcomes. Thus, 96 records were removed through the initial screening, leaving 6 studies, which were all eligible for inclusion upon reading the full text (Figure 1). Three of the studies were conducted by the authors of this review.

### 3.1. Study Characteristics

The included studies were published between 2010 and 2022, and were based on data from women giving birth between 1996 and 2012 (Table 3). Five of the studies focused on women living in northern Europe [40,41,42,43,44] and one was from women in the United States [45]. The study size ranged from 336 [45] to 2374 participants [41]. Five studies were prospective cohort studies [40,42,43,44,45], and one was based on data from a natural experiment, in which contaminated drinking water caused serum PFHxS, PFOS, and PFOA concentrations to be 135, 35, and 5 times higher, respectively, in one Swedish municipality, Ronneby, compared to a nearby municipality [41]. The median duration of total breastfeeding ranged from 6 [45] to 9 months [44]. One study [42] did not consider exclusive breastfeeding, and one study [41] did not give the duration of exclusive breastfeeding. The median duration of exclusive breastfeeding in the other four studies is provided in Table 3, and ranged from 0.07 [45] to 5 months [44]. However, the definition of exclusive breastfeeding was not uniform across the studies. For example, one study relied on the American Academy of Pediatrics guidelines and equaled the duration of exclusive breastfeeding to the infant’s age at the first ever use of formula, water, juice or solid foods [45], while another study defined the duration of exclusive breastfeeding in accordance with the Danish health and food authorities, as the infant’s age when receiving more than one non-breastmilk meal per week [43].

**Figure 2:**
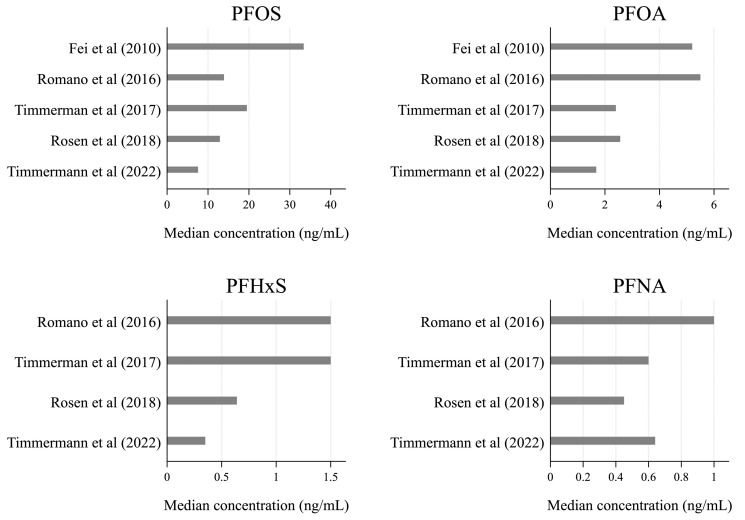
Median serum concentrations of the four most frequently reported types of PFAS: PFOS, PFOA, PFHxS, and PFNA [40,42,43,44,45].

Information about breastfeeding was collected through questionnaires [42,43,44], standardized interviews [40,44,45], health charts [41,43], and text messages [43]. PFAS exposure was assessed based on residential address in one study (a proxy for drinking water exposure to PFAS) [41], while the other five studies relied on PFAS concentrations measured in maternal serum samples collected from gestational week four to two weeks after delivery. Serum PFAS concentrations varied greatly across the study population (Figure 2), but in all five studies, PFOS was found in higher concentrations than the other types of PFAS, with the median serum concentrations ranging from 7.56 to 33.4 ng/mL.

The choice of statistical analysis models differed across the studies. Five of the six studies used Cox proportional hazards models, two studies used logistic regression models, two studies used modified Poisson regression with robust standard errors, and one study used linear regression models (Table 4). Due to the differences in the data collection methods, statistical analyses, and reporting of the results across the studies, this review reports results using hazard ratios (HR), relative risks (RR), and odds ratios (OR), and for the exclusive and total duration of breastfeeding, as well as the initiation of breastfeeding, depending on the available information.

### 3.2. Study Risk of Bias Assessment

Using the ROBINS-E risk of bias analysis, all studies included in this review were considered to be of high quality and have only a low risk of bias, except for concerns about residual confounding (Table 5). Importantly, all six studies considered the risk of confounding from previous breastfeeding, though different approaches were used to control for such confounding. Three studies [42,43,45] obtained information about previous breastfeeding and took this into account in the analyses. Furthermore, all six studies either stratified the analyses by parity, or included an interaction term between PFAS and parity in the statistical models to identify the differential effects between women who had possibly previously breastfed and women who had not. Likewise, all studies adjusted for the variables known to impact breastfeeding duration, such as maternal age, BMI, and education/income.

One study relied on exposure data from a natural experiment [41], and the bias structure in this study was, therefore, different. Although previous breastfeeding is likely to have lowered the serum PFAS concentrations among exposed women, it would not have affected the residential address, which was used as a proxy for exposure in the study by Nielsen et al. Thus, including multiparous women will not overestimate the association in this study. However, some non-differential misclassification and, thus, bias towards the null is likely when exposure assessment is based on a proxy measure of exposure, such as residential address. Multiparous women will likely have lower serum PFAS concentrations than their nulliparous neighbors. Including multiparous women in the high-exposed group might, therefore, lower the median PFAS concentrations in this group and, thus, dilute the association between PFAS exposure and breastfeeding. Accordingly, Nielsen et al. found stronger associations between PFAS exposure and early breastfeeding termination when restricting the analyses to nulliparous women only [41].

Rosen et al. (2018) used combined data from two prior nested case-control studies, resulting in an over-representation of women with subfecundity and preeclampsia, which were both associated with serum PFAS concentrations and decreased breastfeeding duration in the study [42]. However, the authors adjusted for prior study status in their analyses, which should reduce the risk of selection bias based on over-representation [42].

Although PFAS are highly persistent, some imprecision is expected when measuring PFAS in serum at different timepoints during pregnancy and shortly after birth. However, the imprecision is expected to be unrelated to the duration of breastfeeding. Likewise, the women in most studies were unaware of their serum PFAS concentration when breastfeeding, and any misclassification of breastfeeding duration would, thus, be non-differential and most likely lead to bias towards the null.

### 3.3. Breastfeeding Initiation

Two studies examined the role of PFAS in breastfeeding initiation (Table 6). Nielsen et al. (2022) reported PFAS exposure to be associated with a 2.37 (95% confidence interval (CI): 0.84; 6.69) times higher risk of not initiating breastfeeding [41]. In addition, they found that the association between PFAS exposure and the risk of terminating breastfeeding was stronger in early lactation compared to when the child was older [41]. Rosen et al (2018) included only women who initiated breastfeeding in their study, but assessed initiation in a sensitivity analysis and found no statistically significant associations between serum PFAS concentrations and breastfeeding initiation [42].

### 3.4. Exclusive Breastfeeding

Five studies examined the associations between PFAS and exclusive breastfeeding (Table 6), and the Swedish and American studies found no clear associations [41,45]; however, the American study had a very small window of exclusive breastfeeding in which to make comparisons (interquartile range 0.03–0.8 month) [45]. Timmermann et al. (2022) found an 8% (95% CI: 2; 14%) lower risk of breastfeeding termination with each doubling of serum PFHxS concentrations [43], while Fei et al. (2010) found a 37% increased hazard of terminating exclusive breastfeeding at any given time among women in the highest PFOS (95% CI: 14; 64%) and PFOA (95% CI: 12; 69%) quartile compared to those in the lowest quartile [40]. Timmerman et al. (2017) found that each doubling of PFOS, PFOA, and PFDA concentration was associated with a 0.3 (95% CI: 0.1; 0.6), 0.5 (95% CI: 0.3; 0.7), and 0.5 (95% CI: 0.0; 0.4) month shorter duration of exclusive breastfeeding, respectively (Table 6) [44]. When restricting the analyses to primiparous women, most of the mentioned associations were markedly weakened [40,43,44], except for PFDA, for which a doubling in serum concentrations was associated with a 0.5 (95% CI: 0.1; 0.9) month shorter duration of exclusive breastfeeding among primiparous Faroese women [44].

### 3.5. Total Breastfeeding

All six publications contributed data to our study through analyses of total breastfeeding and PFAS comparisons. Among Swedish women, higher exposure to a PFAS mixture in drinking water was associated with a 32% (95% CI: 6; 63%) higher hazard of terminating breastfeeding at 3 months [41], and among Danish women, each doubling in total serum PFAS concentration was associated with a 23% (95% CI: 8; 40%) higher hazard of terminating breastfeeding at any given time [43]. When restricting the analyses to primiparous women, the association in the Danish study was not substantially changed [43], while the association in the Swedish study was strengthened [41] (Table 6).

**Figure 3:**
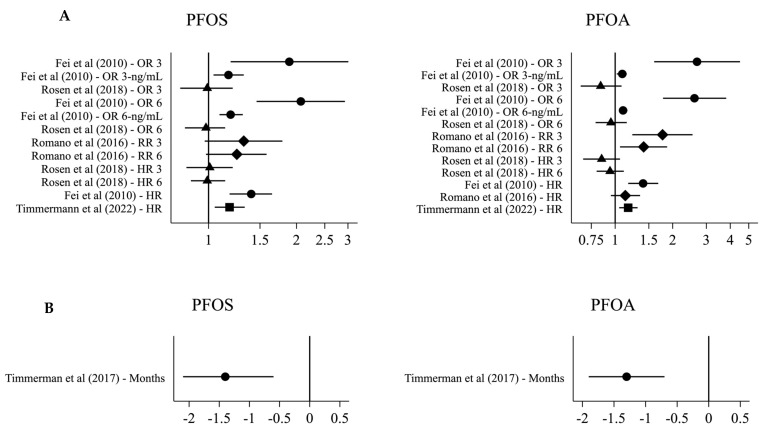
Effect estimates for the association between serum PFOS/PFOA and breastfeeding. (**A**) OR 3/6: odds ratio for termination by 3/6 months for the highest compared to the lowest PFAS quartile Fei et al. (2010) [40], or with an interquartile range difference in PFAS Rosen et al. (2018) [42]. OR 3/6-ng/mL: odds ratio for termination by 3/6 months for each 1 ng/mL PFOS/10 ng/mL PFOA in-crease. RR 3/6: relative risk of termination by 3 months for the highest compared to the lowest PFAS quartile. HR 3/6: hazard ratio for termination at 3/6 months with an interquartile range dif-ference in PFAS. HR: HR for terminating breastfeeding for the highest compared to the lowest PFAS quartile Fei et al. (2010) [40], or with each doubling of PFAS Timmermann et al. (2022) [43] and Romano et al. (2016) [45]. (**B**) months difference in breastfeeding duration with each doubling of PFAS.

For specific types of PFAS, five studies examined the association between PFOS, PFOA, and breastfeeding, and four of these studies found decreased duration of breastfeeding with higher PFOS and PFOA concentrations (Figure 3). Fei et al. (2010) found a 40% increased hazard of terminating breastfeeding at any given time among Danish women in the highest PFOS (95% CI: 18; 65%) and PFOA (95% CI: 17; 68%) quartile compared to those in the lowest quartile, but the associations were weakened when restricting the analyses to primiparous women [40] (Table 6). Romano et al. (2016) found a 77% (95% CI: 23; 154%) increased risk of terminating breastfeeding before 3 months among American women in the highest compared to the lowest PFOA quartile, and the association persisted when restricting the analysis to primiparous women [45]. Slightly weaker associations were seen for PFOS [45]. Among Faroese women, each doubling in PFOS and PFOA concentration was associated with 1.4 (95% CI: 0.6; 2.1) and 1.3 (95% CI: 0.7; 1.9) months, respectively, shorter duration of breastfeeding, and the associations were not substantially weaker among primiparous women [44]. Finally, among Danish women, each doubling in serum PFOS and PFOA concentration during pregnancy was associated with an 18% (95% CI: 5; 33%) and 17% (95% CI: 5; 31%), respectively, higher hazard of terminating breastfeeding at any given time, with associations persisting among primiparous women [43].

PFHxS was examined in four studies (Table 6). Three studies found reduced breastfeeding duration with increasing serum PFHxS concentrations [43,44,45], while one found longer breastfeeding duration with higher serum PFHxS concentrations among Norwegian women [42]. However, all confidence intervals were wide, and none of these findings reached statistical significance (Table 6).

PFNA was likewise examined in four studies, and while the hazard of terminating breastfeeding at 3 months was reduced by 23% (95% CI: 7; 37%) with an interquartile range increase in PFNA among Norwegian women [42], a doubling in PFNA was associated with 1.3 (95% CI: 0.7; 2.0) months shorter duration of breastfeed among Faroese women [44], a 17% (95% CI: 4; 31%) increased hazard of terminating breastfeeding at any given time among Danish women [43], and a 12% (95% CI: −19; 53%) increased risk of terminating breastfeeding before 3 months, among American women [45].

Three studies examined the association between PFDA and breastfeeding. One study found a 27% (95% CI: 14; 38%) lower hazard for breastfeeding cessation at 3 months with each interquartile range increase in PFDA [42], while another study found a 1.3 (95% CI: 0.7; 2.0) month shorter breastfeeding duration with each doubling of PFDA serum concentration [44], and one study found no association [43].

One study additionally examined PFHpS, and the longer carbon chain PFAS PFUnDA, PFDoDA, and PFTrDA. They found increased serum concentrations to be associated with a decreased risk of breastfeeding cessation, though confidence intervals were mostly wide [42].

## 4. Discussion

We systematically describe here, the evidence for associations of several legacy PFAS with the duration of breastfeeding in women from five countries. The majority of the reviewed studies found associations between higher maternal serum PFAS measures and the shorter total duration of breastfeeding, with PFOS, PFOA, and PFNA showing the most consistent associations. Associations between higher maternal serum PFAS measures and the reduced duration of exclusive breastfeeding were seen in a few studies, but the findings were not consistent across the studies. There are many consistent reports on the transfer of PFAS in milk to the infant [27,46,47,48] and the health effects thereof [25,49,50], but this review confirms that there are also effects on breastfeeding, potentially limiting the amount and quality of maternally-derived nutrition provided to the infant. Breast milk is a critical source of immunoglobulins and other proteins and fats needed to support the development of the infant’s nervous and immune system [2,3,51], and the infant may develop deficits in these systems because of PFAS exposure [52,53,54,55,56]. It has thus, become imperative to protect women of reproductive age and their infants from PFAS exposures.

Associations similar to those found in this review have been observed for other environmental chemicals, the pesticide metabolite dichlorodiphenyldichloroethylene (DDE) was linked to the shorter duration of breastfeeding in the 1980s and 90s [57,58], and, more recently, maternal Bisphenol A (BPA) exposure has also been linked to the reduced duration of breastfeeding, although the imprecision in the BPA studies did not allow for definitive conclusions [59,60].

Studies in mice also observe causal relationships between the exposure to some PFAS during pregnancy and adverse lactation outcomes. In pregnant mice, 5 mg/kg/d of PFOA exposure during pregnancy delayed differentiation of the lactating gland with subsequent growth restriction and increased neonatal mortality observed in pups [35,61], making them less likely to meet developmental milestones. Histopathological and molecular evaluations of the mammary gland, during the period of lactation, suggested that PFOA inhibited differentiation of the mammary epithelial cells and altered the expression of milk proteins in the gland, consistent with disrupted lactogenesis. Furthermore, studies in multiple strains of mice exposed during pregnancy to doses lower than 5 mg PFOA/kg/d revealed developmental abnormalities of the mammary epithelium in their female pups that persisted into adulthood [62,63,64] and were noted in multiple generations in CD-1 mice [61]. Together these studies suggest that the breast epithelium is a target for PFOA-induced toxicities. Recent studies on a PFOA replacement chemical, hexafluoropropylene oxide-dimer acid (HFPO-DA, commonly called GenX), similarly caused reduced mouse pup weight at postnatal day 5 [65] or postnatal days 1–21, following prenatal exposures [66]. Whether the differentiation of the mammary gland of their lactating dams were impacted by the exposure has yet to be reported for GenX.

There are potentially at least two important aspects of lactation that may be impacted by PFAS exposure, the proper differentiation of the epithelium (addressed above) and adequate endocrine function needed to support lactation. Despite consistencies between the experimental and epidemiological findings for a PFAS exposure-associated effect on lactation, relatively little is known about the potential mechanisms by which PFAS might affect breastfeeding. Prenatal PFAS exposure has been associated with lower prolactin concentrations in female infants [67], and in pregnant mice, exposure to PFOS significantly reduced the serum concentrations of prolactin-family hormones [68]. However, no such association was detected among pregnant women [43], though the timing of potential effects of PFAS on prolactin hormones during pregnancy remains unclear. Activation of the peroxisome proliferator-activated receptor (PPAR) *α* during pregnancy can impair mammary lobuloalveolar development in mice [69], and since the PPAR*α* is activated by some PFAS [70], these chemicals could potentially affect mammary gland development and, thus, breastfeeding through activation of the PPAR*α* There are numerous proteins known to be imperative to adequate milk production that have not been investigated as molecular targets of PFAS exposures (e.g., ErbB3, Akt, Stat5A [71]). Prolactin receptors, critical to proper lactation, are known to be regulated by numerous endocrine and mammary-made hormones shown to be altered by PFAS in other female reproductive tissues [24,72]. Finally, thyroid dysfunction is known to promote lactation deficits in rodent models [73], and thyroid hormones are consistently reported as altered by PFAS in women and rodents [74]. Recent work involving rats suggests that PFOS-induced decreases in systemic thyroid hormones may trigger expression of factors altering trafficking, metabolism, and excretion of the thyroid hormone, and that the pituitary and thyroid may not be the targets [75]. As the breast possesses these capabilities, they may be viable mechanisms to pursue in future work.

In this study, we found the associations between the PFAS measures and the total duration of breastfeeding were stronger than those seen for PFAS and exclusive breastfeeding, and there are a couple of scenarios that might explain the difference. The assessment of total duration of breastfeeding through a questionnaire have been shown to be more reliable than assessment of exclusive breastfeeding by questionnaire [76], biasing estimates for exclusive breastfeeding more towards the null. The definition of exclusive breastfeeding is also not consistent across the studies, and studies with a strict definition had a very short median duration of exclusive breastfeeding making it harder to detect differences related to PFAS exposure. It is also possible that compared to early weaning, the decision to use nutritional supplementation is affected by factors unrelated to insufficient milk supply, such as maternal commitments outside the home. These factors are most likely unrelated to PFAS exposure, and the imprecision caused by not taking these factors into account will, thus, likely increase bias towards the null.

Among the six studies included in this review, five found associations between PFAS and reduced breastfeeding duration, while one study [42] found no such associations or even opposite effects. Based on the described methods, we cannot identify any obvious explanations for the differences in the results. Rosen et al. (2018) suggested that the different findings between theirs and other studies might be explained by differences in maternal serum PFAS concentrations [42]. However, when compared to the other studies, including those published after the Rosen et al. study, differences in the exposure concentrations do not seem to explain the differences.

The strengths of this review include that all six studies were prospective (ensuring temporality), of high quality, and had only a low risk of bias. Thus, all studies took previous breastfeeding into account, either directly or by stratifying by parity. Also, the majority of the studies adjusted for education, income, or other markers of socioeconomic status, which might be important, as socioeconomic status is associated with breastfeeding duration [77] and might also, in some settings, be associated with PFAS exposure [78].

An additional strength is that this review and the conclusions drawn from the six identified studies relied on studies with different designs. When assessing associations between PFAS exposure and breastfeeding duration, the risk of confounding from previous breastfeeding is likely the strongest cause of bias. Using a proxy measure for PFAS, as was conducted in the Swedish study [41], can eliminate this kind of bias, but at the cost of increasing the exposure measurement error [79]. Increased non-differential exposure measurement error will most likely lead to underestimation, while confounding from previous breastfeeding will lead to overestimation of the true association. Thus, when studies with different study designs and different bias structures find similar results, it is less likely that the findings are caused by bias [80].

A limitation of this review is that comparisons across the studies included in the review were impaired by the studies relying on different statistical methods, with some studies presenting ORs, while others presented RRs or HRs. As breastfeeding termination is not a rare outcome, the ORs will be higher than the RRs would have been using the same data, and the size of the effect estimates are not directly comparable across the studies. Despite these obvious differences, we included the estimates in joint figures allowing comparison of the direction of the effect estimates. Another limitation is that the six studies did not all examine the same set of PFAS. PFHpS, PFUnDA, PFDoDA, and PFTrDA were examined in only one study, whereas PFOS and PFOA were examined in five studies, making it easier to identify consistencies in PFOS and PFOA associations across the studies. An additional potential limitation is that we identified only six studies that examined the role of PFAS on breastfeeding, and that all studies were from either northern Europe or North America, and only one study included high-exposed women. Additional studies are needed from other parts of the world, including Asia, Africa, and South America, and studies from highly polluted areas would be of particular interest. Regardless, these data demonstrating consistent positive associations that are replicated across studies in different populations, with different study designs, reduce the likelihood that specific biases or potential confounders in individual studies explain the positive associations, especially when much of the bias is likely towards the null.

In the five studies relying on PFAS measurements in maternal blood samples, samples were obtained at various timepoints during pregnancy or in the early post-partum period. PFAS are highly persistent chemicals and, under normal circumstances, serum concentration will not change much over a year [81]. However, during pregnancy, maternal blood volume increases, and serum PFAS concentrations subsequently decrease [82]. The studies that collected blood samples at different timepoints did take the timing of blood sampling into account in their analyses; thus, we suspect that the timing of the exposure assessments did not severely affect the results.

As with all systematic reviews, we cannot dismiss the risk of publication bias, and it is possible that additional null-finding studies have not been published, which could have impacted our findings. PFAS have been getting more attention from research scientists, communities that have been shown to be highly exposed, public health professionals, and regulatory agencies [83,84]. By placing more focus on this topic of research, we hope to increase the body of literature examining the association between PFAS and breastfeeding duration. In fact, regulatory agencies examining experimental evidence are beginning to appreciate the effects of PFAS on the health and function of the breast, yet the National Academies of Sciences, Engineering, and Medicine did not consider duration of lactation in their 2022 “Guidance on PFAS Exposure, Testing, and Clinical Follow-Up”, and they chose adults as the population for proposed health guidance instead of infants [85]. At present, based on experimental studies in rodents, the development of the mammary gland is considered the most sensitive developmental outcome of PFOA exposure [22] and has been considered for regulatory action in the U.S. [86].

The benefits of breastfeeding are well known, and for decades, health authorities have worked to promote breastfeeding by providing education and support to new mothers and the health professionals around them [87]. However, little emphasis has been given to environmental exposures that might impair breastfeeding, the functional aspect of the breast. This review suggests that breastfeeding may be sensitive to PFAS exposures, thus, emphasizing that breastfeeding is vulnerable to these environmental pollutants. Thus, in addition to the direct negative effects of PFAS on child health [31,32], maternal PFAS exposure may also indirectly negatively impact child health through reduced breastfeeding. Given that other environmental and lifestyle factors also affect women’s ability to breastfeed [60,88,89], more research is needed to elucidate which exposures might impair breastfeeding and to better understand the mechanisms behind such effects.

## Figures and Tables

**Table 1:** Search strategy.

**Facet 1**	PFAS Perfluoroalkyl substances Polyfluoroalkyl substances PFOS PFOA
**Facet 2**	Breastfeeding (Title) Breast Feeding (Mesh/key term)
**Restrictions**	English language
**Exclusions**	Reviews
**PubMed Search**	(“PFAS” OR “Perfluoroalkyl Substances” OR “polyfluoroalkyl substances” OR “PFOS” OR “PFOA”) AND (“Breastfeeding” [Title] OR “Breast Feeding” [Mesh]) NOT (Review [Publication Type]) Filter: “English”
**Embase (Ovid) Search**	(1) PFAS.af. (2) Perfluoroalkyl Substances.af. (3) Polyfluoroalkyl substances.af. (4) PFOS.af. (5) PFOA.af. (6) Breastfeeding.m_titl. (7) exp breast feeding/ (8) 1 or 2 or 3 or 4 or 5 (9) 6 or 7 (10) 8 and 9 (11) limit 10 to English language (12) limit 11 to “review” (13) 11 not 12

**Table 2:** PECO (population, exposure, comparator, and outcomes) statement.

**Population**	Human: Pregnant women or new mothers
**Exposure**	Per- and polyfluoroalkyl substance (PFAS) exposure
**Comparator**	Increasing exposure or high versus low exposure
**Outcomes**	Total duration of breastfeeding Duration of exclusive breastfeeding Breastfeeding initiation

**Table 3:** Studies included in the review.

First Author (Publication Year)	Country	Study Design	N	PFAS Concentrations (Median, ng/mL)	Breastfeeding Duration (Median, Months)
Fei et al. (2010) [40]	Denmark	Prospective cohort study: Danish National Birth Cohort with data collection in years 1996–2002.	1346	PFOS: 33.4 PFOA: 5.2	Total: 7.9 Exclusive: 3.9
Romano et al. (2016) [45]	USA	Prospective cohort study: The Health Outcomes and Measures of the Environment study with data collection in years 2003–2006.	336	PFOS: 13.9 PFOA: 5.5 PFHxS: 1.5 PFNA: 1.0	Total: 6 ^1^ Exclusive: 0.07 ^1^
Timmerman et al. (2017) [44]	Faroe Islands	Prospective cohort study: Two birth cohorts with data collection in years 1997–2000 and 2007–2009.	987	PFOS: 19.5 PFOA: 2.4 PFHxS: 1.5 PFNA: 0.6 PFDA: 0.3	Total: 9 Exclusive: 5
Rosen et al. (2018) [42]	Norway	Prospective cohort study: Norwegian Mother and Child Cohort Study with data collection in years 1999–2008.	1716	PFOS: 12.9 PFOA: 2.56 PFHxS: 0.64 PFNA: 0.45 PFDA: 0.09 PFHpS: 0.14 PFUnDA: 0.19 PFDoDA: 0.04 PFTrDA: 0.04	Total: > 6 ^2^ Exclusive: NA
Nielsen et al. (2022) [41]	Sweden	Natural experiment (contamination of drinking water): Municipality of Ronneby plus reference group from nearby municipality, children born in years 1999–2009.	2374	NA ^3^	Total: 6–7 Exclusive: NI
Timmermann et al. (2022) [43]	Denmark	Prospective cohort study: Odense Child Cohort with data collection in years 2010–2012.	925	PFOS: 7.56 PFOA: 1.68 PFHxS: 0.35 PFNA: 0.64 PFDA: 0.29 ∑PFAS: 10.86	Total: 7.6 Exclusive: 2.5

NA: not assessed. NI: no information. ^1^ Among breastfeeding initiators only (81.3% of the population). ^2^ The women were not followed beyond 6 months, but only 21% had ceased breastfeeding at that time. ^3^ PFAS concentrations were not measured in the study population, but in Ronneby, women of childbearing age (21–40 years old) had geometric mean PFHxS, PFOS, and PFOA concentrations of 60, 80, and 5 ng/mL, respectively. Corresponding concentrations in the reference population were 0.9, 3.2, and 1.3 ng/mL, respectively.

**Table 4:** Analytical methods.

First Author (Publication Year)	Statistical Analysis Model	Effect Estimate
Fei et al. (2010) [40]	Cox regression + logistic regression	(1) HR of terminating breastfeeding for the highest compared to the lowest PFAS quartile (2) OR of weaning before 3 and 6 months/terminating exclusive before 1 and 4 months for the highest compared to the lowest PFAS quartile (3) OR of weaning before 3 and 6 months/terminating exclusive before 1 and 4 months per 1 ng/mL PFOS/10 ng/mL PFOA
Romano et al. (2016) [45]	Poisson regression with robust standard errors + Cox regression	(1) HR of terminating breastfeeding (before 12 months) with each doubling in PFOA (2) RR of weaning by 3 and 6 months/terminating exclusive breastfeeding by 3 months for the highest compared to the lowest PFAS quartile
Timmerman et al. (2017) [44]	Linear regression	(1) Months difference in breastfeeding duration with each doubling in PFAS
Rosen et al. (2018) [42]	Cox regression with censoring at 3 or 6 months + logistic regression. Excluded women who never initiated breastfeeding.	(1) HR of breastfeeding cessation up until 3 and 6 months with an interquartile range difference in PFAS (all except PFDoDA and PFTrDA)/concentrations > vs. ≤ LOQ (PFDoDA and PFTrDA) (2) OR of breastfeeding cessation before 3 and 6 months with an interquartile range difference in PFAS
Nielsen et al. (2022) [41]	Modified Poisson regression with robust error variance + Cox regression with censoring at 6/12 months for exclusive/total breastfeeding	(1) RR of not initiating breastfeeding and not achieving exclusive/total breastfeeding by 3/6 months for high-exposed compared to reference group (2) - HR of terminating exclusive breastfeeding before 6 months for high-exposed compared to reference group - Time-varying HR of terminating total breastfeeding before 12 months for high-exposed compared to reference group
Timmermann et al. (2022) [43]	Cox regression. Women terminating breastfeeding for reasons not related to insufficient lactation or with no reason given were censured.	(1) HR of terminating breastfeeding with each doubling in PFAS concentration

HR: hazard ratio. OR: odds ratio. RR: risk ratio.

**Table 5:** Study risk of bias assessment.

First Author (Publication Year)	Bias Due to Confounding		Bias Arising from Measurement of the Exposure	Bias in Selection of Participants	Bias Due to Post-Exposure Interventions	Bias Due to Missing Data	Bias Arising from Measurements of the Outcome	Bias in Selection of the Reported Results	Overall Risk of Bias
	Confounding from Previous Breastfeeding	Other Confounding							
Fei et al. (2010) [40]	Low risk. Stratification by parity.	Low risk. Adjusted for parity, maternal age at delivery, pre-pregnancy body mass index, maternal socioeconomic status, alcohol consumption, and smoking during pregnancy, and gestational age at blood drawing.	Low risk. Blood samples, GA 4–14 weeks.	Low risk. Selection was not affected by the exposure or outcome.	Low risk. Participants were unaware of their exposure.	Low risk. Few data were missing and controlled for.	Low risk of bias towards null. Interviews at 6 and 18 months after birth.	Low risk. Multiple analyses were performed, but all estimates were provided.	Low
Romano et al. (2016) [45]	Low risk. Adjustment for total duration of breastfeeding previous children and stratification by parity.	Low risk. Adjusted for parity, maternal age, race/ethnicity, marital status, household income, maternal serum cotinine during pregnancy, alcohol use during pregnancy, and gestational week at blood draw.	Low risk of bias towards null. Blood samples, GA 16 weeks (85%), 26 weeks (10%), or delivery (5%).	Low risk. Selection was not affected by the exposure or outcome.	Low risk. Participants were unaware of their exposure.	Low risk. The proportion of missing data was most likely similar across groups.	Low risk of bias towards null. Standardized surveys by phone every 3 months until breastfeeding was discontinued or the child’s third birthday.	Low risk. Multiple analyses were performed, but all estimates were provided.	Low
Timmerman et al. (2017) [44]	Low risk. Tested interaction between PFAS and parity.	Low risk. Adjusted for parity, cohort, maternal age, pre-pregnancy BMI, pregnancy alcohol intake, pregnancy smoking, education, and employment.	Low risk of bias towards null. Blood samples, GA 34–36 weeks (older cohort) or two weeks after their term date (younger cohort).	Low risk. Selection was not affected by the exposure or outcome.	Low risk. Participants were unaware of their exposure.	Low risk. Few data were missing and controlled for.	Low risk of bias towards null. Questionnaire followed by an interview at 18 months (only younger cohort) and 5 years after birth.	Low risk. Multiple analyses were performed, but all estimates were provided.	Low
Rosen et al. (2018) [42]	Low risk. Adjustment for previous breastfeeding duration and stratification by parity.	Low risk. Adjusted for parity, maternal age at birth, smoking during pregnancy, pre-pregnancy BMI, and prior study status.	Low risk. Blood samples GA 17–20 weeks.	Low risk. Combined data from two prior nested case-control studies. Selection was not affected by the exposure or outcome.	Low risk. Participants were unaware of their exposure.	Low risk. Missing data were imputed using the Markov chain Monte Carlo method.	Low risk of bias towards null. Self-reported questionnaire 6 months after delivery.	Low risk. Multiple analyses were performed, but all estimates were provided.	Low
Nielsen et al. (2022) [41]	Low risk. Use of residential address (and stratification by parity).	Low risk. Adjusted for parity, maternal age, BMI, education level, country of birth, and tobacco use during pregnancy.	Low risk of bias towards null. Residential addresses of mothers during the 5-year window before delivery.	Low risk. Participants were selected based on residential address. Selection was not related to the outcome.	Low risk. Participants were aware of their exposure risk, but all were advised to breastfeed.	Low risk. Few data were missing.	Moderate risk of bias towards null. Child health care charts. Information on breastfeeding and formula feeding was available monthly during the first year and less frequently thereafter.	Low risk. Multiple analyses were performed, but all estimates were provided.	Low
Timmermann et al. (2022) [43]	Low risk. Tested interaction between PFAS and parity.	Low risk. Adjusted for education, body mass index, national origin, and expectations of giving formula, parity, smoking, duration of previous breastfeeding, and previous inadequate lactation.	Low risk. Blood samples, median GA 12 weeks (5th–95th percentiles, 10–16 weeks).	Low risk. Selection was not affected by the exposure or outcome.	Low risk. Participants were unaware of their exposure.	Low risk. The proportion of missing data was most likely similar across groups.	Low risk of bias towards null. Self-reported questionnaire 3 and 18months post-partum. Weekly text messages from a subgroup of women. Additional information from health visitor contacts for early weaners.	Low risk. Multiple analyses were performed, but all estimates were provided.	Low

GA: gestational age.

**Table 6:** Summary of study findings.

First Author (Publication Year)	Total Breastfeeding (All)	Total Breastfeeding (Primiparous)	Exclusive Breastfeeding (All)	Exclusive Breastfeeding (Primiparous)	Author Conclusion
Fei et al. (2010) [40]	PFOS: HR: 1.40 (1.18; 1.65) OR 3: 1.89 (1.19; 3.01) OR 6: 2.07 (1.46; 2.93) OR 3-ng/mL: 1.17 (1.04; 1.32) OR 6-ng/mL: 1.19 (1.09; 1.31) PFOA: HR: 1.40 (1.17; 1.68) OR 3: 2.68 (1.60; 4.50) OR 6: 2.60 (1.78; 3.81) OR 3-ng/mL: 1.09 (1.02; 1.15) OR 6-ng/mL: 1.10 (1.05; 1.16)	PFOS: HR: 1.17 (0.90; 1.52) OR 3: 1.24 (0.62–2.46) OR 6: 1.52 (0.89; 2.60) OR 3-ng/mL: 1.12 (0.94; 1.34) OR 6-ng/mL: 1.20 (1.04; 1.37) PFOA: HR: 0.98 (0.69; 1.38) OR 3: 1.82 (0.66; 5.07) OR 6: 1.50 (0.74; 3.06) OR 3-ng/mL: 1.02 (0.93; 1.10) OR 6-ng/mL: 1.03 (0.97; 1.10)	PFOS: HR: 1.37 (1.14; 1.64) OR 1: 1.55 (0.89; 2.71) OR 4: 1.86 (1.24; 2.79) OR 1-ng/mL: 1.09 (0.93; 1.27) OR 4-ng/mL: 1.14 (1.02; 1.27) PFOA: HR: 1.37 1.12–1.69 OR 1: 2.19 1.16; 4.14 OR 4: 2.59 1.66; 4.04 OR 1-ng/mL: 1.05 (0.98; 1.13) OR 4-ng/mL: 1.06 (1.00; 1.12)	PFOS: HR: 1.23 (0.93; 1.64) OR 1: 0.78 (0.35; 1.70) OR 4: 1.19 (0.66; 2.15) OR 1-ng/mL: 0.98 (0.78; 1.23) OR 4-ng/mL: 1.04 (0.89; 1.22) PFOA: HR: 1.16 0.81; 1.66 OR 1: 0.55 0.21;1.42 OR 4: 0.91 0.43;1.89 OR 1-ng/mL: (0.93 0.82; 1.06) OR 4-ng/mL: (0.98 (0.91; 1.06)	These findings suggest that PFOA and PFOS may reduce the ability to lactate, but could equally reflect reverse causation since no association was seen in primiparous women.
Romano et al. (2016) [45]	PFOS:	PFOS:	PFOS:		Maternal serum PFOA concentrations were inversely related to the duration of any breastfeeding. Maternal serum PFAS concentrations were not associated with exclusive breastfeeding.
	RR 3: 1.32 (0.97; 1.79)	RR 3: 1.21 (0.71; 2.08) ^1^	RR 3: 0.98 (0.87; 1.10)		
	RR 6: 1.25 (0.98; 1.58)	RR 6: 1.07 (0.70; 1.61) ^1^			
		
	PFOA:	PFOA:	PFOA:		
	HR: 1.13 (0.95; 1.35)	RR 3: 2.17 (1.17; 4.01) ^1^	RR 3: 1.12 (0.98; 1.28)		
	RR 3: 1.77 (1.23; 2.54)	RR 6: 1.41 (0.85; 2.32) ^1^			
	RR 6: 1.41 (1.06; 1.87)				
		
	PFHxS:		PFHxS:		
	RR 3: 1.39 (0.99; 1.96)		RR 3: 0.94 (0.84; 1.06)		
	RR 6: 1.22 (0.96; 1.55)				
		
	PFNA:		PFNA:		
	RR 3: 1.12 (0.81; 1.53)		RR 3: 0.96 (0.85; 1.08)		
	RR 6: 1.13 (0.90; 1.43)				
Timmerman et al. (2017) [44]	PFOS months: −1.4 (−2.1; −0.6) PFOA months: −1.3 (−1.9; −0.7) PFHxS months: −0.2 (−0.5; 0.2) PFNA months: −1.3 (−2.0; −0.7) PFDA months: −0.8 (−1.4; −0.3)	PFOS months: −1.3 (−2.3; −0.3) PFOA months: −1.1 (−2.0; −0.1) PFHxS months: −0.1 (−0.5; 0.3) PFNA months: −1.6 (−2.6; −0.6) PFDA months: −1.6 (−2.6; −0.7)	PFOS months: −0.3 (−0.6; −0.1) PFOA months: −0.5 (−0.7; −0.3) PFHxS months: −0.1 (−0.2; 0.1) PFNA months: −0.2 (−0.5; −0.0) PFDA months: −0.2 (−0.4; 0.0)	PFOS months: −0.2 (−0.6; 0.1) PFOA months: −0.4 (−0.8; 0.0) PFHxS months: −0.0 (−0.1; 0.1) PFNA months: −0.3 (−0.8; 0.1) PFDA months: −0.5 (−0.9; −0.1)	Increased maternal serum PFAS concentrations are associated with a decreased duration of breastfeeding.
Rosen et al. (2018) [42]	PFOS:	PFOS:			Unexpected observation of inverse associations of PFNA, PFDA, and PFUnDA with breastfeeding cessation. A positive association of PFOS with breastfeeding was observed when accounting for other exposures. No association between PFOA and breastfeeding was observed.
	HR 3: 1.01 (0.84; 1.21)	HR 3: 0.94 (0.77; 1.16)			
	HR 6: 0.99 (0.87; 1.14)	HR 6: 0.94 (0.81; 1.09)			
	OR 3: 0.99 (0.80; 1.21)				
	OR 6: 0.98 (0.83; 1.14)				
	
	PFOA:	PFOA:			
	HR 3: 0.85 (0.68; 1.06)	HR 3: 0.84 (0.67; 1.04)			
	HR 6: 0.94 (0.80; 1.11)	HR 6: 0.91 (0.78; 1.06)			
	OR 3: 0.84 (0.66; 1.08)				
	OR 6: 0.95 (0.79; 1.15)				
	
	PFHxS:	PFHxS:			
	HR 3: 0.88 (0.75; 1.03)	HR 3: 0.93 (0.78; 1.11)			
	HR 6: 0.92 (0.82; 1.03)	HR 6: 0.96 (0.84; 1.09)			
	OR 3: 0.86 (0.72; 1.03)				
	OR 6: 0.91 (0.80; 1.05)				
	
	PFNA:	PFNA:			
	HR 3: 0.77 (0.63; 0.93)	HR 3: 0.72 (0.58; 0.88)			
	HR 6: 0.84 (0.73; 0.97)	HR 6: 0.81 (0.69; 0.95)			
	OR 3: 0.73 (0.59; 0.91)				
	OR 6: 0.83 (0.70; 0.98)				
	
	PFDA:	PFDA:			
	HR 3: 0.73 (0.62; 0.86)	HR 3: 0.69 (0.58; 0.82)			
	HR 6: 0.82 (0.72; 0.92)	HR 6: 0.78 (0.68; 0.89)			
	OR 3: 0.70 (0.58; 0.84)				
	OR 6: 0.80 (0.69; 0.93)				
	
	PFHpS:	PFHpS:			
	HR 3: 0.96 (0.81; 1.14)	HR 3: 0.95 (0.77; 1.16)			
	HR 6: 0.96 (0.85; 1.08)	HR 6: 0.96 (0.83; 1.11)			
	OR 3: 0.96 (0.79; 1.15)				
	OR 6: 0.96 (0.83; 1.11)				
	
	PFUnDA:	PFUnDA:			
	HR 3: 0.73 (0.62; 0.86)	HR 3: 0.68 (0.57; 0.81)			
	HR 6: 0.81 (0.72; 0.93)	HR 6: 0.78 (0.68; 0.90)			
	OR 3: 0.70 (0.58; 0.84)				
	OR 6: 0.79 (0.68; 0.91)				
	
	PFDoDA:	PFDoDA:			
	HR 3: 0.97 (0.68; 1.41)	HR 3: 0.97 (0.64; 1.46)			
	HR 6: 0.90 (0.69; 1.17)	HR 6: 0.88 (0.65; 1.19)			
	OR 3: 0.97 (0.65; 1.45)				
	OR 6: 0.88 (0.65; 1.19)				
	
	PFTrDA:	PFTrDA:			
	HR 3: 0.99 (0.69; 1.40)	HR 3: 0.87 (0.58; 1.30)			
	HR 6: 0.98 (0.76; 1.26)	HR 6: 0.98 (0.74; 1.29)			
	OR 3: 0.97 (0.66; 1.43)				
	OR 6: 0.97 (0.72; 1.29)				
Nielsen et al. (2022) [41]	∑PFAS: RR initiation: 2.37 (0.84; 6.69) RR 6: 1.17 (0.07–1.40) HR 0: 1.58 (1.12; 2.22) HR 3: 1.32 (1.06; 1.63) HR 6: 1.09 (0.93; 1.29) HR 10: 0.86 (0.65; 1.14)	∑PFAS: RR initiation: 2.92 (0.34; 23.01) RR 6: 1.56 (1.16; 2.11) HR 0: 2.49 (1.50; 4.14) HR 3: 1.82 (1.32; 2.51) HR 6: 1.33 (1.05; 1.69) HR 10: 0.88 (0.59; 1.30)	∑PFAS: RR 3: 1.12 (0.92; 1.37) HR: 1.14 (0.92; 1.40)	∑PFAS: RR 3: 1.21 (0.92; 1.61) HR: 1.30 (0.96; 1.76)	Exposure to high levels of PFAS seemed to be associated with increased risks of not initiating breastfeeding and shorter breastfeeding duration in primiparous women. The findings imply that the ability of first-time mothers to establish breastfeeding is a sensitive outcome after high exposure to PFAS.
Timmermann et al. (2022) [43]	PFOS HR: 1.18 (1.05; 1.33) PFOA HR: 1.17 (1.05; 1.31) PFHxS HR: 1.05 (0.96; 1.14) PFNA HR: 1.17 (1.04; 1.31) PFDA HR: 1.02 (0.93; 1.12) ∑PFAS HR: 1.23 (1.08; 1.40)	PFOS HR: 1.15 (1.00; 1.33) PFOA HR: 1.19 (1.04; 1.37) PFHxS HR: 1.04 (0.93; 1.16) PFNA HR: 1.18 (1.02; 1.36) PFDA HR: 1.03 (0.91; 1.16) ∑PFAS HR: 1.21 (1.03; 1.43)	PFOS HR: 0.94 (0.85; 1.04) PFOA HR: 1.02 (0.93; 1.12) PFHxS HR: 0.92 (0.86; 0.98) PFNA HR: 0.91 (0.82; 1.01) PFDA HR: 0.97 (0.89; 1.06) ∑PFAS HR: 0.94 (0.84; 1.05)	PFOS HR: 0.95 (0.84; 1.08) PFOA HR: 0.98 (0.87; 1.12) PFHxS HR: 0.94 (0.85; 1.03) PFNA HR: 0.89 (0.78; 1.02) PFDA HR: 0.97 (0.86; 1.09) ∑PFAS HR: 0.94 (0.81; 1.09)	Higher serum PFAS concentrations were associated with a shorter duration of breastfeeding.

HR: hazard ratio for terminating breastfeeding for the highest compared to the lowest PFAS quartile Fei et al. (2010) [40], or with each doubling of PFAS Timmerman et al. (2017) [44] and Romano et al. (2016) [45]. HR 3/6/10: hazard ratio for termination at 3/6/10 month with an interquartile range difference in PFAS Rosen et al. (2018) [42], or for high-exposed compared to the reference population Nielsen et al. (2022) [41]. OR 3/6: odds ratio for termination by 3/6 months for the highest compared to the lowest PFAS quartile Fei et al. (2010) [40], or with an interquartile range difference in PFAS Rosen et al. (2018) [42]. OR 3/6-ng/mL: odds ratio for termination by 3/6 months for each 1 ng/mL PFOS/10 ng/mL PFOA increase. RR 3/6: relative risk of termination by 3/6 months for the highest compared to the lowest PFAS quartile Romano et al. (2016) [45]], or for high-exposed compared to the reference group Nielsen et al. (2022) [41]. ^1^ RR estimates used to create supplemental Figure 1 were provided directly by the author. RR initiation: relative risk of not initiating breastfeeding for high-exposed compared to the reference group. PFAS months: months difference in breastfeeding duration with each doubling of PFAS. RR initiation: relative risk of not initiating for high-exposed compared to the reference group.

## Data Availability

Data can be made available upon request.
